# Alcohol’s Role in Gastrointestinal Tract Disorders

**Published:** 1997

**Authors:** Christiane Bode, J. Christian Bode

**Affiliations:** Christiane Bode, Ph.D., is professor and chief of the Section of Physiology of Nutrition (140), Hohenheim University, Stuttgart, Germany. J. Christian Bode, M.D., is professor of medicine and chief of the Section of Gastroenterology, Hepatology and Endocrinology in the Department of Internal Medicine, Robert-Bosch-Krankenhaus, Stuttgart, Germany

**Keywords:** ethanol metabolism, AODE (alcohol and other drug effects), mouth, esophagus, stomach, intestine, gastric mucosa, intestinal mucosa, gastric lesion, gastric acid, gastrointestinal function, gastrointestinal absorption, muscle, neoplastic disease, toxins, free radicals, etiology, literature review

## Abstract

When alcohol is consumed, the alcoholic beverages first pass through the various segments of the gastrointestinal (GI) tract. Accordingly, alcohol may interfere with the structure as well as the function of GI-tract segments. For example, alcohol can impair the function of the muscles separating the esophagus from the stomach, thereby favoring the occurrence of heartburn. Alcohol-induced damage to the mucosal lining of the esophagus also increases the risk of esophageal cancer. In the stomach, alcohol interferes with gastric acid secretion and with the activity of the muscles surrounding the stomach. Similarly, alcohol may impair the muscle movement in the small and large intestines, contributing to the diarrhea frequently observed in alcoholics. Moreover, alcohol inhibits the absorption of nutrients in the small intestine and increases the transport of toxins across the intestinal walls, effects that may contribute to the development of alcohol-related damage to the liver and other organs.

Among the many organ systems that mediate alcohol’s effects on the human body and its health, the gastrointestinal (GI) tract plays a particularly important part. Several processes underlie this role. First, the GI tract is the site of alcohol absorption into the bloodstream and, to a lesser extent, of alcohol breakdown and production. (For more information on alcohol absorption, metabolism, and production in the GI tract, see sidebar, pp. 82–83.) Second, the direct contact of alcoholic beverages with the mucosa^1^ that lines the upper GI tract can induce numerous metabolic and functional changes. These alterations may lead to marked mucosal damage, which can result in a broad spectrum of acute and chronic diseases, such as acute gastrointestinal bleeding (from lesions in the stomach or small intestine) and diarrhea. Third, functional changes and mucosal damage in the gut disturb the digestion of other nutrients as well as their assimilation into the body, thereby contributing to the malnutrition and weight loss frequently observed in alcoholics. Fourth, alcohol-induced mucosal injuries—especially in the upper small intestine—allow large molecules, such as endotoxin and other bacterial toxins, to pass more easily into the blood or lymph. These toxic substances can have deleterious effects on the liver and other organs.

**Alcohol Absorption, Metabolism, and Production in the Gastrointestinal Tract**

The excessive consumption of alcoholic beverages interferes with the normal function and structure of the gastrointestinal (GI) tract. The relationship between alcohol and the GI tract is a two-way street, however, and the GI tract plays a role in the absorption, metabolism, and production of alcohol.

***Absorption***

Throughout the GI tract, alcohol absorption into the bloodstream occurs through a process called simple diffusion. The rate at which this process occurs depends on several factors, primarily the difference between the alcohol concentrations in the GI organs and in the adjacent small blood vessels, the regional blood flow, and the permeability of the GI tract lining (i.e., the mucosa) in question. For example, the higher the concentration of the ingested alcohol, the more alcohol the mucosa absorbs. In contrast, the presence of food in the stomach decreases the rate of alcohol absorption. Other factors that may affect alcohol absorption include the type of alcoholic beverage, the drinker’s gender and body temperature, the presence of certain medications in the body, and the types of spices in the food (Bode 1980). For example, alcohol absorption occurs more slowly after the ingestion of beer than after the ingestion of an equal amount of alcohol in the form of whisky or brandy. Most of these factors probably inhibit or enhance alcohol absorption by affecting the movement of the stomach muscles (i.e., gastric motility) and small intestinal blood flow.

**Figure:**
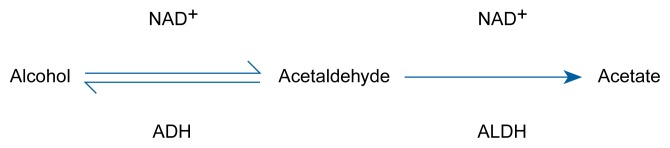
The pathway of alcohol metabolism. Once in the liver, alcohol is converted into acetaldehyde, and the acetaldehyde is converted into acetate. The enzyme alcohol dehydrogenase (ADH) assists the chemical reaction in (i.e., catalyzes) the first half of alcohol metabolism, and the enzyme aldehyde dehydrogenase (ALDH) catalyzes the second half. NAD^+^ is a coenzyme that plays an accessory role in enzyme catalysis.

***Metabolism***

More than two decades ago, researchers found that the initial breakdown of alcohol (i.e., first-pass metabolism) occurs not only in the liver, as with most other nutrients, but also in the stomach and the small intestine (Mezey 1985). The enzyme alcohol dehydrogenase (ADH), which mediates the first step of alcohol degradation (see figure), is present in the mucosa of the stomach and the small intestine. In fact, several ADH variants (i.e., isoenzymes) with different kinetic properties exist in the mucosa of the GI tract; these isoenzymes permit alcohol metabolism over a wide range of concentrations. The ADH isoenzyme pattern in the GI tract differs from that found in the liver.

Some researchers have postulated that first-pass metabolism in humans occurs primarily in the stomach (Gentry et al. 1994) and correlates significantly with gastric ADH activity. However, other investigators have questioned the stomach’s role in first-pass alcohol metabolism (Levitt 1994). The proportion of alcohol eliminated by gastric first-pass metabolism also remains controversial (Sato and Kitamura 1996); compared with hepatic alcohol degradation, gastric first-pass metabolism seems to be quantitatively important only at low alcohol concentrations. In women, first-pass metabolism is less efficient than in men. Consequently, women achieve higher blood alcohol concentrations than men with the same low gastric alcohol concentration because more alcohol passes from a woman’s stomach into the small intestine, where it is absorbed into the bloodstream. Furthermore, gastric first-pass metabolism decreases with long-term alcohol consumption, partly because of diminished ADH activity (Gentry et al. 1994).

A recent study suggests that alcohol also can be metabolized by bacteria residing in the large intestine (Salaspuro 1996). In this pathway, alcohol is transported to the colon via the bloodstream and converted to acetaldehyde by bacterial ADH (see figure). The acetaldehyde subsequently can be metabolized further by the enzyme aldehyde dehydrogenase (ALDH), which is localized in the colonic mucosa or the colonic bacteria. Alternatively, the acetaldehyde can be absorbed into the bloodstream and transported to the liver for further degradation. Because ALDH activity in the colonic mucosa is low, acetaldehyde accumulates in the colon and may even exceed the concentration found in the liver (Salaspuro 1996). These high acetaldehyde levels in the colon may contribute to the development of alcohol-induced diarrhea and—after absorption into the blood—liver injury.

***Production***

In many animal species, including humans, alcohol is not only degraded but also produced in the GI tract. This alcohol production is a by-product of the bacterial breakdown of ingested carbohydrates. Alcohol also is formed in the human stomach, and in patients with disturbed gastric emptying, the concentrations can be as high as 0.35 percent (i.e., about four times as high as the blood alcohol levels for intoxication) (Bode and Bode 1992). Two factors favor gastric alcohol production in these patients. First, they produce less gastric acid and thus allow the proliferation of bacteria in the stomach. Second, the patients retain their food in the stomach for an extended period of time. Both factors lead to an increase in the bacterial degradation of nutrients and thus an increase in alcohol production.

— Christiane Bode and J. Christian Bode

ReferencesBodeJCAlcohol and the gastrointestinal tractAdvances in Internal Medicine and Pediatrics451751980700254010.1007/978-3-642-67632-1_1BodeJCBodeCAlcohol malnutrition and the gastrointestinal tractWatsonRRWatzlBNutrition and AlcoholBoca Raton, FLCRC Press1992403428GentryRTBaraonaELieberCSAgonist: Gastric first pass metabolism of alcoholJournal of Laboratory and Clinical Medicine123212619948288957LevittMDAntagonist: The case against first-pass metabolism of ethanol in the stomachJournal of Laboratory and Clinical Medicine123283119948288958MezeyEEffect of ethanol on intestinal morphology, metabolism, and functionSeitzHKKommerellBAlcohol Related Diseases in GastroenterologyBerlinSpringer-Verlag1985342360SalaspuroMBacteriocolonic pathway for ethanol oxidation: Characteristics and implicationsAnnals of Medicine281952001996881116210.3109/07853899609033120SatoNKitamuraTFirst-pass metabolism of ethanol: An overviewGastroenterology111114311501996883161210.1016/s0016-5085(96)70085-9

Over the past three decades, researchers have made major progress toward understanding alcohol’s many acute and chronic effects on GI-tract function and structure.

This article reviews some of these findings, focusing primarily on insights gained during the past 10 years. (For extensive reviews of the developments in this field up to the early 1980’s, see Beazell and Ivy 1940; Bode 1980).

## The GI Tract—An Overview

**Figure 1:**
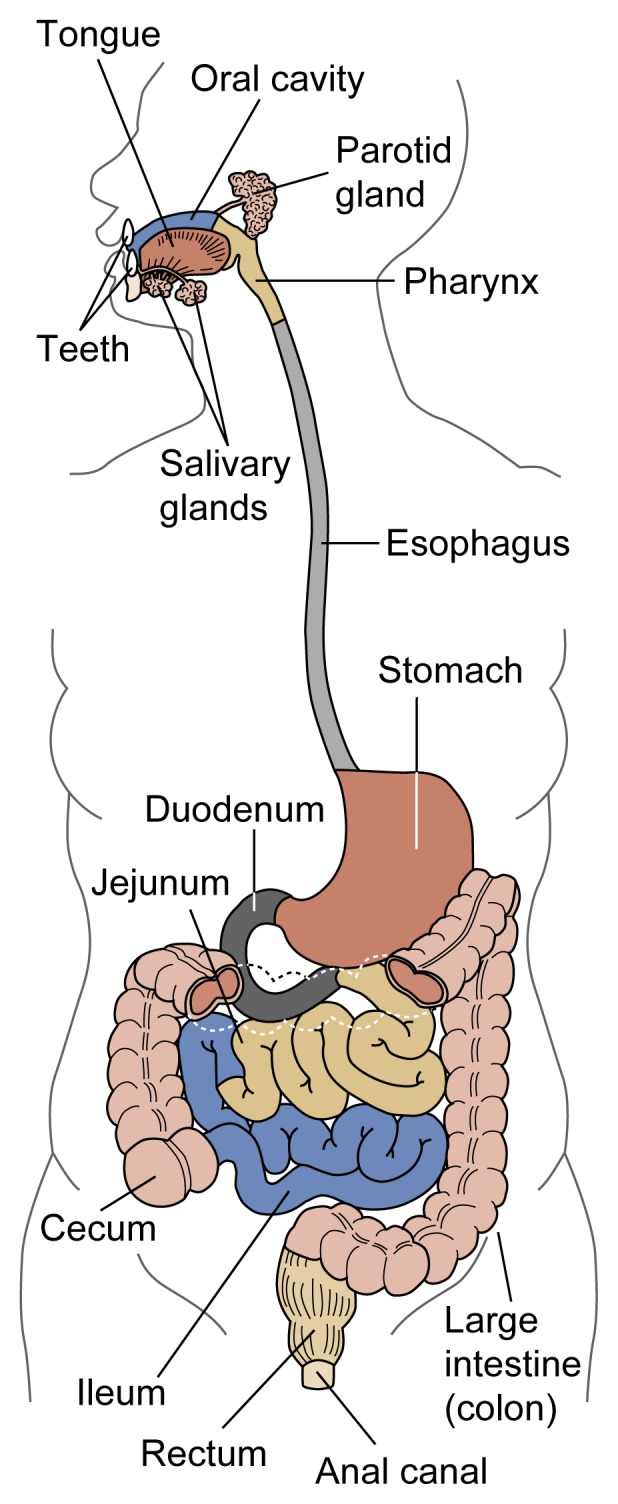
Schematic representation of the human gastrointestinal tract. The small intestine comprises the duodenum, the ileum, and the jejunum.

The GI tract’s functions are to physically and chemically break down ingested food, allow the absorption of nutrients into the bloodstream, and excrete the waste products generated. The GI tract can be viewed as one continuous tube extending from the mouth to the anus (figure 1), which is subdivided into different segments with specific functions.

In the mouth, or oral cavity, the teeth mechanically grind the food into small pieces. Moreover, saliva excreted by the salivary glands initiates the food’s chemical degradation. From the oral cavity, the food passes through the throat (i.e., pharynx) into the esophagus. The coordinated contraction and relaxation of the muscles surrounding the esophagus propels the food into the stomach.

In the stomach, the chemical degradation of the food continues with the help of gastric acid and various digestive enzymes. Excessive gastric acid production can irritate the mucosa, causing gastric pain, and result in the development of gastric ulcers. Two bands of muscle fibers (i.e., sphincters) close off the stomach to the esophagus and the intestine. Weakness of the sphincter separating the stomach from the esophagus allows the stomach content to flow back into the esophagus. This process, which is called gastroesophageal reflux, can lead to heartburn as well as inflammation (i.e., reflux esophagitis) and even to the development of ulcers in the lower part of the esophagus.

**Figure 2:**
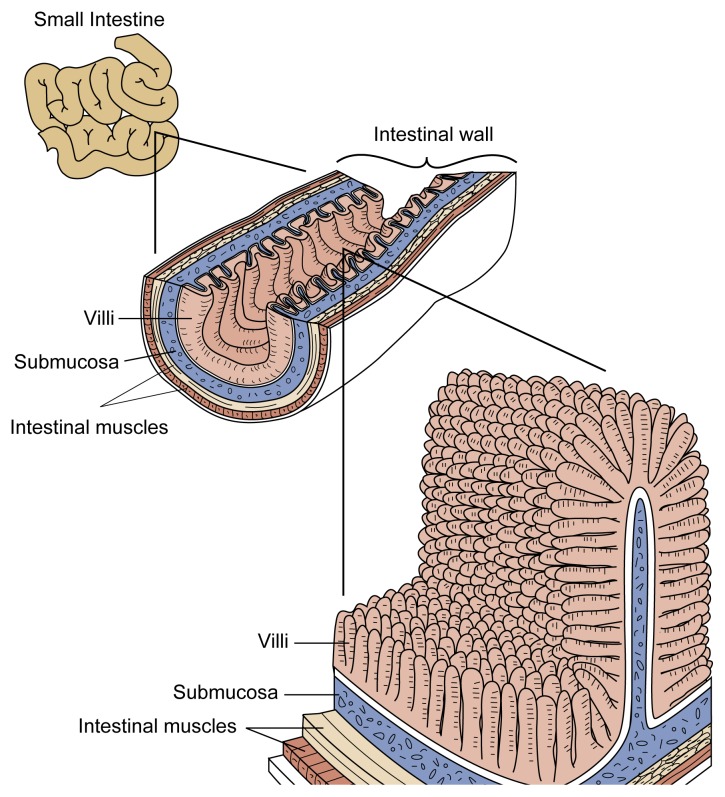
Schematic illustration of the villi lining the small intestine. These villi serve to increase the internal surface area of the intestine and thus enhance the absorption of nutrients.

From the stomach, the food enters the small intestine, which is divided into three segments: the duodenum, the jejunum, and the ileum. Like the esophagus and stomach, the intestine is surrounded by layers of muscles, the rhythmic movements of which help mix the food mass and push it along the GI tract. The intestine’s inner mucosal surface is covered with small projections called villi, which increase the intestinal surface area (figure 2). As the food mass moves through the small intestine, digestive enzymes secreted by the intestinal cells complete the chemical degradation of nutrients into simple molecules that can be absorbed through the intestinal wall into the bloodstream. What finally remains in the intestine are primarily indigestible waste products. These products progress into the large intestine, where the waste is compacted and prepared for excretion through the anus. Like the small intestine, the large intestine can be divided into three segments: the cecum; the colon, which constitutes about 80 percent of the large intestine; and the rectum. The following sections review alcohol’s effect on the different regions of the GI tract.

## The Oral Cavity and the Esophagus

The oral cavity, pharynx, esophagus, and stomach are exposed to alcohol immediately after its ingestion. Thus, alcoholic beverages are almost undiluted when they come in contact with the mucosa of these structures. It is therefore not surprising that mucosal injuries (i.e., lesions) occur quite frequently in people who drink large amounts of alcohol.^2^

Chronic alcohol abuse damages the salivary glands and thus interferes with saliva secretion. In alcoholics this damage commonly manifests itself as an enlargement (i.e., hypertrophy) of the parotid gland, although the mechanisms leading to this condition are unknown. Moreover, alcoholics may suffer from inflammation of the tongue (i.e., glossitis) and the mouth (i.e., stomatitis). It is unclear, however, whether these changes result from poor nutrition or reflect alcohol’s direct effect on the mucosa. Finally, chronic alcohol abuse increases the incidence of tooth decay, gum disease, and loss of teeth (Kranzler et al. 1990).

Alcohol consumption can affect the esophagus in several ways. For example, alcohol distinctly impairs esophageal motility, and even a single drinking episode (i.e., acute alcohol consumption) significantly weakens the lower esophageal sphincter. As a result, gastroesophageal reflux may occur, and the esophagus’ ability to clear the refluxed gastric acid may be reduced. Both of these factors promote the occurrence of heartburn. Moreover, some alcoholics exhibit an abnormality of esophageal motility known as a “nutcracker esophagus,” which mimics symptoms of coronary heart disease (Bode and Bode 1992).^3^

Chronic alcohol abuse leads to an increased incidence not only of heartburn but also of esophageal mucosal inflammation (i.e., esophagitis) and other injuries that may induce mucosal defects (i.e., esophagitis with or without erosions). In addition, alcoholics make up a significant proportion of patients with Barrett’s esophagus. This condition, which occurs in 10 to 20 percent of patients with symptomatic gastroesophageal reflux disease (Wienbeck and Berges 1985), is characterized by changes in the cell layer lining the esophagus (i.e., the epithelium) that lead to abnormal acid production. A diagnosis of Barrett’s esophagus is an important indicator of an increased risk of esophageal cancer, because in some patients the altered epithelial cells become cancerous.

Another condition affecting alcoholics is Mallory-Weiss syndrome, which is characterized by massive bleeding caused by tears in the mucosa at the junction of the esophagus and the stomach. The syndrome accounts for 5 to 15 percent of all cases of bleeding in the upper GI tract. In 20 to 50 percent of all patients, the disorder is caused by increased gastric pressure resulting from repeated retching and vomiting following excessive acute alcohol consumption (Bode and Bode 1992).

## The Stomach

Both acute and chronic alcohol consumption can interfere with stomach functioning in several ways. For example, alcohol—even in relatively small doses—can alter gastric acid secretion, induce acute gastric mucosal injury, and interfere with gastric and intestinal motility.

### Gastric Acid Secretion

Analyses in several animal species found that acute alcohol administration by mouth or by direct infusion into the stomach (i.e., intragastrically) or the blood (i.e., intravenously) can affect gastric acid secretion (Chari et al. 1993). The secretory response of the stomach varies considerably, however, depending on the species studied and the alcohol concentrations used. In healthy, nonalcoholic humans, intravenous alcohol administration of 0.3 to 0.5 grams per kilogram (g/kg) body weight or intragastric infusion of alcohol solutions with concentrations of up to 5 percent stimulate gastric acid secretion, whereas intragastric infusion of higher concentrations has either no effect or a mildly inhibitory one (Chari et al. 1993). Accordingly, alcoholic beverages with a low alcohol content (e.g., beer and wine) strongly increase gastric acid secretion and the release of gastrin, the gastric hormone that induces acid secretion. In contrast, beverages with a higher alcohol content (e.g., whisky and cognac) stimulate neither gastric acid secretion nor gastrin release.

The mechanisms underlying the effects of alcoholic beverages on gastric acid secretion have not yet been identified. Alcohol may interact directly with the gastric mucosa (i.e., topical stimulation); or, it may act through a more general mechanism affecting the release of hormones and the regulation of nerve functions involved in acid secretion (Chari et al. 1993). Moreover, researchers have shown that after beer consumption, gastric acid secretion also is stimulated by by-products of the fermentation process other than alcohol (Chari et al. 1993).

Chronic alcohol abuse also affects gastric function. Thus, alcoholics have a significantly higher incidence of shrinkage (i.e., atrophy) of the gastric mucosa and decreased gastric secretory capacity than do healthy control subjects of comparable age and sex (Bode and Bode 1992). The resulting decrease in acid production reduces the stomach’s ability to destroy the bacteria that enter with food and thus favors the colonization of the upper small intestine with potentially harmful microorganisms. Abstinence, however, can at least partly reverse these changes.

### Acute Gastric Mucosal Injury

Researchers have known for more than 100 years that alcohol abuse can cause mucosal inflammation (for a review, see Beazell and Ivy 1940). In addition, alcohol abuse is an important cause of bleeding (i.e., hemorrhagic) gastric lesions that can destroy parts of the mucosa. Although low or moderate alcohol doses do not cause such damage in healthy subjects, even a single episode of heavy drinking can induce mucosal inflammation and hemorrhagic lesions. Nonsteroidal anti-inflammatory drugs (e.g., aspirin and ibuprofen) may aggravate the development of alcohol-induced acute gastric lesions.

How alcohol damages the gastric mucosa has not yet been determined. Studies in both animals and humans have found that alcohol concentrations of 10 percent and more disrupt the gastric mucosal barrier and increase the mucosa’s permeability (Bode and Bode 1992). The changes induced by short-term exposure to alcoholic beverages are rapidly reversible. Prolonged alcohol exposure, however, disturbs the microcirculation and leads to progressive structural mucosal damage.

Several studies have suggested that the decreased formation of hormone-like substances called prostaglandins might play a role in alcohol-induced mucosal injury (Bode et al. 1996). Prostaglandins protect the gastric mucosa from damage by agents such as aspirin that break the gastric mucosal barrier without inhibiting acid secretion. Other studies have indicated that an alcohol-dependent increase in the production of leukotrienes—compounds produced by the immune system that cause allergic and inflammatory reactions—also might contribute to the development of alcohol-induced mucosal injury (Bode and Bode 1992).

### Gastric and Intestinal Motility

Alcohol can interfere with the activity of the muscles surrounding the stomach and the small intestine and thus alter the transit time of food through these organs. In humans, alcohol’s effect on gastric motility depends on the alcohol concentration and accompanying meals. In general, beverages with high alcohol concentrations (i.e., above 15 percent) appear to inhibit gastric motility and thus delay the emptying of the stomach. As a result of the increased gastric transit time, bacterial degradation of the food may begin; the resulting gases may lead to feelings of fullness and abdominal discomfort.

In the small intestine, alcohol decreases the muscle movements that help retain the food for further digestion (i.e., the impeding wave motility). In contrast, alcohol does not affect the movements that propel food through the intestine (i.e., the propulsive wave motility) in either alcoholics or healthy subjects. These effects may contribute to the increased sensitivity to foods with a high sugar content (e.g., candy and sweetened juices), shortened transit time, and diarrhea frequently observed in alcoholics (Bode and Bode 1992).

## The Small Intestine

As described previously, the small intestine is the organ in which most nutrients are absorbed into the bloodstream. Studies in humans and animals as well as in tissue culture have demonstrated that alcohol can interfere with the absorption of several nutrients. Alcohol itself, however, also is rapidly absorbed in the small intestine. In the human jejunum, for example, the alcohol concentration can drop from 10 percent to just 1.45 percent over a distance of only 30 centimeters (12 inches, about a quarter of the total length of the jejunum) (Bode 1980). Therefore, alcohol’s effects on nutrient absorption may vary throughout the small intestine, and tissue-culture experiments with constant alcohol concentrations may not always reflect the conditions in the body.

Studies in laboratory animals have demonstrated that acute alcohol consumption can inhibit the absorption of water, sodium, glucose, and certain amino acids and fatty acids in the small intestine (Bode 1980; Mezey 1985). Several studies in humans have analyzed the effects of chronic alcohol consumption with the following results:

- Both in healthy people and in alcoholics, chronic alcohol consumption led to markedly reduced water and sodium absorption in the jejunum and ileum (Bode and Bode 1992; Pfeiffer et al. 1992).
- Alcoholics exhibited a reduced absorption of carbohydrates, proteins, and fats in the duodenum, but not in the jejunum (Pfeiffer et al. 1992) (see table).
- Alcoholics without confounding disorders, such as cirrhosis or impaired pancreatic function, exhibited malabsorption of fat and protein (see table).
- Alcoholics showed malabsorption of xylose, a sugar frequently used to study the function of the digestive tract. The proportion of alcoholics who experienced this malabsorption ranged from 18 to 76 percent in various studies (see table). This variation may reflect differences in the nutritional status, the mean daily alcohol intake, or the presence of alcohol-related liver disease among the studies’ subjects.
- After chronic alcohol consumption, the absorption of thiamine (vitamin B_1_), folic acid, and vitamin B_12_ was either unchanged or decreased (Mezey 1985; Bode and Bode 1992). Folic acid deficiency, which frequently occurs in alcoholics, can result in various disorders of the GI tract as well as in anemia. However, this deficiency is more likely to result from a diet containing insufficient folic acid than from poor folic acid absorption (Halstedt and Keen 1990).

**Table:** Summary of Studies on Malabsorption of Carbohydrates, Fat, and Protein in Alcoholics Without Cirrhosis or Obvious Pancreatic Insufficiency

A. Frequency of Abnormal Absorption Among Alcoholics		

Nutrient Studied	Number of Studies	% Subjects With Abnormal Absorption
		
D-Xylose	5	18–76
Fat^a^	2	35–56
Protein^b^	1	52

**B. Mean Decrease in Absorption Compared With Nonalcoholic Controls**^c^		

**Nutrient Studied**		**% Decrease**
		
Carbohydrates		45
Lipids		40
Protein		81

a Fat absorption was determined by measuring fat excretion in the feces.

b Protein absorption was determined by measuring nitrogen excretion in the feces.

c The study measured the absorption of a nutrient solution in the duodenum.

SOURCES: A: Bode and Bode 1992; B: Pfeiffer et al. 1992.

In summary, alcohol inhibits absorption of a variety of nutrients. The importance of these absorption disorders in the development of nutritional disturbances in alcoholics, however, is unclear. In alcoholics with limited pancreatic function or advanced liver disease, digestion of nutrients may be a more significant problem than impaired absorption disorders.

### Intestinal Enzymes

Alcohol can interfere with the activity of many enzymes that are essential for intestinal functioning. One of these enzymes is lactase, which breaks down the milk sugar lactose; lactase deficiency results in lactose intolerance. Alcohol also interferes with some of the enzymes involved in transporting nutrients from the intestine into the bloodstream and inhibits important enzymes that participate in the metabolism of drugs and other foreign organic substances in the gut (for reviews, see Mezey 1985; Bode 1980).

### Intestinal Mucosal Injury

Excessive alcohol consumption frequently causes mucosal damage in the upper region of the duodenum. Even in healthy people, a single episode of heavy drinking can result in duodenal erosions and bleeding. Animal studies have indicated that several mechanisms contribute to the development of these mucosal injuries (Ray et al. 1989) (for a review, see Bode and Bode 1992). First, alcohol can directly disturb the integrity of the mucosal epithelium. Second, alcohol induces the release of noxious signaling molecules, such as cytokines, histamine, and leukotrienes. These substances can damage the small blood vessels, or capillaries, in the intestinal mucosa and induce blood clotting. Such clotting may lead to an impaired transport of fluids across the capillaries; fluid accumulation under the tips of the villi; and, eventually, destruction of the tips of the villi. The resulting lesions allow large molecules, such as endotoxins and other bacterial toxins, to enter the bloodstream and the lymph. Third, as in the stomach, decreased prostaglandin synthesis may contribute to changes in the capillaries and to the development of mucosal injury.

### Intestinal Permeability

**Figure 3:**
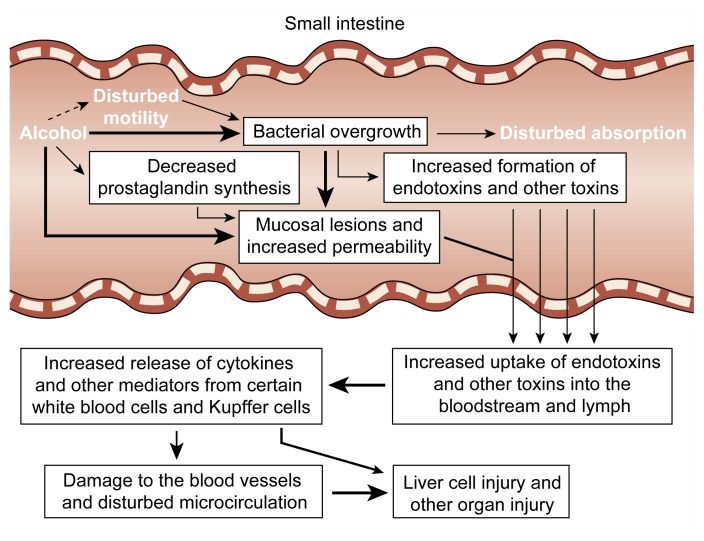
Schematic presentation of the possible causes and consequences of mucosal injury, increased permeability of the intestinal mucosa to macromolecules, and bacterial overgrowth in the small intestine of chronic alcohol abusers. Thickness of arrows indicates strength of association between phenomena. (For definitions, see glossary, pp. 93–96.)

In animal studies, alcohol administration increased the permeability of the intestinal mucosa, allowing large molecules that normally cannot cross the intestinal wall intact (e.g., hemoglobin) to travel between the gut and the bloodstream (Bode 1980). Similarly, intestinal permeability was enhanced in nonintoxicated alcoholics (Bode and Bode 1992). The enhanced permeability induced by acute and chronic alcohol ingestion could allow toxic compounds, such as endotoxin and other bacterial toxins, to enter the bloodstream and subsequently reach the liver. The presence of endotoxin in the blood has been documented in patients with early stages of alcohol-related liver damage and transiently, after excessive alcohol consumption, in people with no evidence of liver disease (Fukui et al. 1991). Endotoxins can induce the release of cytokines (e.g., tumor necrosis factor) and interleukins from certain white blood cells and from Kupffer cells in the liver. These cytokines, in turn, may play a role in the development of alcohol-related damage to the liver and other organs (Khoruts et al. 1991; Schäfer et al. 1995) (figure 3). (For more information on the association of endotoxin and cytokines with liver damage, see the article by Maher, pp. 5–12.)

### Intestinal Bacterial Microflora

Certain bacteria that are a major source of endotoxin may overgrow the normal bacterial flora in the jejunum of alcoholics (Bode and Bode 1992). Together with the altered permeability of the gut induced by alcohol, this process may allow an increased escape of endotoxin from the intestine into the blood vessels leading to the liver, thus increasing the liver’s exposure to these toxins and, consequently, the risk of liver injury (figure 3). The hypothesis that bacterial overgrowth may be responsible for the development of alcohol-related organ damage has been supported by the observation that sterilization of the intestine prevents alcohol-induced liver injury in animal experiments (Adachi et al. 1995).

### The Large Intestine

Until recently, alcohol’s effects on the large intestine had received only minor attention. Studies in dogs found that acute alcohol administration depressed the colon’s impeding motility but enhanced its propulsive motility (Mezey 1985). In healthy humans, alcohol administration also significantly reduced the frequency and strength (i.e., amplitude) of the muscle contractions in a segment of the rectum (Mezey 1985). These effects could reduce the transit time—and thus the compaction—of the intestinal contents and thereby contribute to the diarrhea frequently observed in alcoholics.

## Medical Consequences

Alcohol-induced digestive disorders and mucosal damage in the GI tract can cause a variety of medical problems. These include a loss of appetite and a multitude of abdominal complaints, such as nausea, vomiting, feelings of fullness, flatulence, and abdominal pain. Diseases of the liver and pancreas may contribute to and aggravate these complaints. Thus, about 50 percent of alcoholics with an initial stage of liver damage (i.e., fatty liver) and 30 to 80 percent of patients with an advanced stage of alcohol-induced liver injury (i.e., alcoholic hepatitis) report some symptoms of abdominal discomfort (Bode and Bode 1992). These abdominal complaints can lead to reduced food intake, thereby causing the weight loss and malnutrition commonly observed in alcoholics.

In addition to causing abdominal complaints, alcohol plays a role in the development of cancers of the GI tract. It is likely, however, that alcohol does not cause GI-tract cancers by itself but acts in concert with other cancer-inducing agents (i.e., as a cocarcinogen) (for reviews, see Seitz and Simanowski 1988; Garro and Lieber 1990). Alcohol abuse, like smoking, is associated with the development of cancers of the tongue, larynx (i.e., the organ of voice), and pharynx; both alcohol consumption and smoking independently increase the risk for these tumors (Bode 1980). Epidemiological studies also strongly indicate that chronic alcohol consumption, especially of distilled spirits, markedly contributes to the development of esophageal cancer (Bode 1980; Wienbeck and Berges 1985). Thus, after adjusting for smoking habits, heavy beer drinkers have a 10 times greater risk and heavy whisky drinkers a 25 times greater risk of developing esophageal cancer, compared with people who consume less than 30 g of alcohol (i.e., about 2 standard drinks) daily. The differences between beer and whisky drinkers remain even if they consume the same amount of pure alcohol. In drinkers who also smoke 20 cigarettes or more daily, the risk of esophageal cancer increases about 45-fold (Seitz and Simanowski 1988).

Heavy alcohol consumption also is associated with the development of tumors in the colon and rectum. However, the relative risk of cancer is higher for rectal cancer than for colon cancer. Moreover, the increased risk of rectal cancer appears to result mainly from heavy beer consumption, whereas distilled spirits appear to have no effect.

## Summary

Alcohol consumption can interfere with the function of all parts of the gastrointestinal tract. Acute alcohol ingestion induces changes in the motility of the esophagus and stomach that favor gastroesophageal reflux and, probably, the development of reflux esophagitis. Alcohol abuse may lead to damage of the gastric mucosa, including hemorrhagic lesions. Beverages with a low alcohol content stimulate gastric acid secretion, whereas beverages with a high alcohol content do not.

In the small intestine, alcohol inhibits the absorption of numerous nutrients. The importance of these absorption disorders for the development of nutritional disturbances in alcoholics, however, is unclear. In alcoholics with other digestive disorders (e.g., advanced liver disease or impaired pancreatic function), impaired digestion likely is more significant. Acute alcohol consumption also damages the mucosa in the upper region of the small intestine and may even lead to the destruction of the tips of the villi. The findings of human and animal studies suggest that these mucosal defects favor the following sequence of events: Alcohol-induced mucosal damage in the small intestine increases the mucosa’s permeability, facilitating the transport of large molecules, such as bacterial endotoxin and/or other toxins, into the blood or lymph. This results in the release of potentially toxic cytokines by certain white blood cells and Kupffer cells. These cytokines, in turn, exert multiple injurious effects on membranes and the microcirculation. The result is possible cell damage and even cell death in the liver and other organs.

Motility disorders, maldigestion, and malabsorption in alcoholics can result in digestive problems, such as anorexia, nausea, and abdominal pain. Alcohol abuse also promotes the development of cancers of the tongue, larynx, pharynx, and esophagus. Finally, the results of recent epidemiological studies indicate an association between alcohol consumption and the development of colorectal cancer.

## Footnotes

1 For a definition of this and other technical terms used in this article, see the central glossary, pp. 93–96.

2 The alcohol amount necessary to cause mucosal injury varies significantly among individual drinkers and depends, for example, on whether alcohol consumption occurs on an empty stomach or is accompanied by a meal. Thus, no clear threshold exists above which alcohol exerts its adverse effects. However, the risk for adverse effects such as tissue damage generally increases following the consumption of more than 2 ounces of alcohol, which corresponds to approximately four standard drinks (i.e., “heavy” or “excessive” drinking).

3 The term “nutcracker esophagus” refers to the painful, spasmodic contractions of the esophagus that the patients who suffer the disorder describe as feeling as though the esophagus were being squeezed by a nutcracker.
